# Internal jugular phlebectasia: case report and literature review

**DOI:** 10.1590/1677-5449.202400952

**Published:** 2025-10-20

**Authors:** Letícia Stefani Pacheco, Marcos Antonio Bonacorso Manhanelli, José Augusto Costa

**Affiliations:** 1 Pontifícia Universidade Católica de São Paulo – PUC-SP, Faculdade de Ciência Médicas e da Saúde, Sorocaba, SP, Brasil.; 2 Conjunto Hospitalar de Sorocaba – CHS, Sorocaba, SP, Brasil.

**Keywords:** jugular veins, dilation, vascular ectasia

## Abstract

Internal jugular phlebectasia is a non-tortuous abnormal dilation of the internal jugular vein that presents as a cervical mass with a saccular or fusiform aneurysmal appearance and is most common in male pediatric patients. Its etiologies include congenital, cervical trauma, tumors, and previous surgeries. The clinical picture is generally asymptomatic. In symptomatic cases, the most common complaint is pain in the affected cervical area. Diagnosis is established by visualization of the dilation when triggered by increased intrathoracic pressure during actions such as coughing or crying and is confirmed by imaging studies such as Doppler ultrasound and computed tomography. Therapeutic management is typically conservative, with surgical intervention reserved for cases with symptoms or complications.

## INTRODUCTION

Internal jugular phlebectasia can be described as a rare vascular condition consisting of non-tortuous, anomalous dilation of the internal jugular vein in the supraclavicular region, along the anterior margin of the sternocleidomastoid muscle.^1^

Phlebectasia of the jugular and cervical venous system is a condition that has been described sporadically in the literature, with few descriptions before the 1970s. With advances in major laryngeal and cervical surgery and as noninvasive diagnostic methods have developed, the condition is being recognized with increasing frequency. More than 100 cases of phlebectasia involving the veins of the neck, including the anterior and external jugular veins, have been reported in the global literature to date. This venous aneurysm was documented for the first time in 1928 by Harris and in 1952 Gerwig used the term “phlebectasia” to describe the condition.^2-6^ In Brazil, cases have been reported from 1999 to 2021.^7-9^

The majority of patients are pediatric and male. Clinically, internal jugular phlebectasia is asymptomatic and may be identified as a painless mass with flaccid consistency on the lateral neck, generally unilateral, producing a swelling in the presence of effort that increases intrathoracic pressure.^10,11^

There may be related cervical pain, in addition to other manifestations such as changes to the voice, vocal cord paralysis, and dysphagia. These are the result of the proximity of the vein to the vagus nerve and other cranial nerves, which may be compressed by phlebectasia, causing feelings of tightness, suffocation and vertigo/dizziness, lingual pain, loss of voice when talking out loud, and discomfort when practicing physical activity, swallowing, or coughing.^12,13^

## PART I – CLINICAL CASE

This case report was approved by the Research Ethics Committee with CAAE 75131623.5.0000.5373 and Substantiated Opinion 6.592.364.

A 6-year-old male patient was brought to the emergency room after being in a medium-velocity automobile accident, involving an impact to the right lateral aspect of the head and neck. Immediately after the trauma, he only exhibited pain, which was controlled with analgesia. During the initial physical examination, a right cervical swelling was observed when the child was crying, simulating a Valsalva maneuver. This swelling was painless, transitory, extensive, voluminous, and of soft consistency. The child’s legal guardian reported that there had been no mass or swelling in that area prior to the trauma.

## PART II – MANAGEMENT

**Figure 1:**
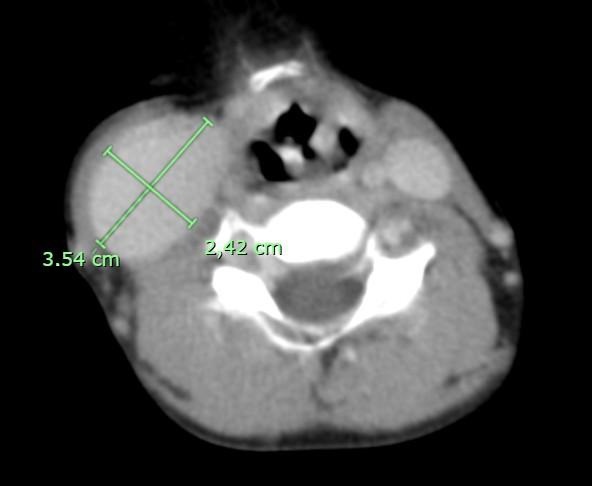
CT image in axial plane with intravenous contrast, showing the right internal jugular vein.

**Figure 2:**
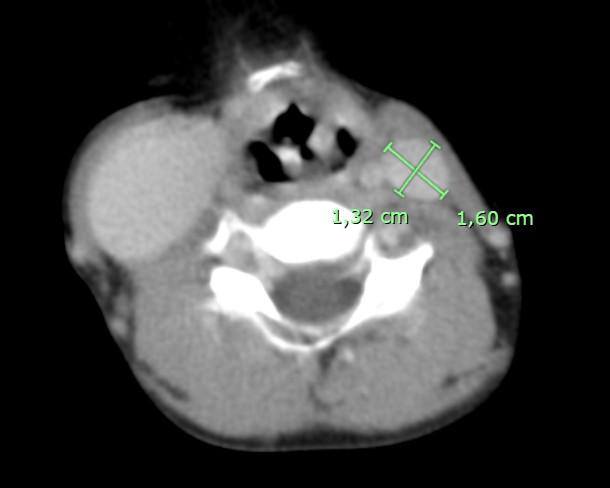
CT image in axial plane with intravenous contrast, showing the left internal jugular vein.

Since the patient had been brought to the emergency room after trauma caused by an automobile accident, a computed tomography (CT) scan of the neck with contrast was ordered (Figures 1 and 2) for initial work-up. This scan confirmed the presence of bilateral phlebectasia of the internal jugular vein, more accentuated on the right. However, artifacts caused by movement compromised analysis of the slices.

After admission to the hospital, Doppler ultrasonography was conducted, showing the right internal jugular vein compressible, patent, with flow in phase with breathing, and with no echogenic content or signs of pathological reflux. The CT images showed a dilatation of the right internal jugular vein (Figure 1), with an anteroposterior diameter of 2,42 cm and a lateral-lateral diameter of 3,54 cm, and another dilatation of the left internal jugular vein (Figure 2) with an anteroposterior diameter of 1,60 cm by a lateral-lateral diameter of 1,32 cm.

**Figure 3:**
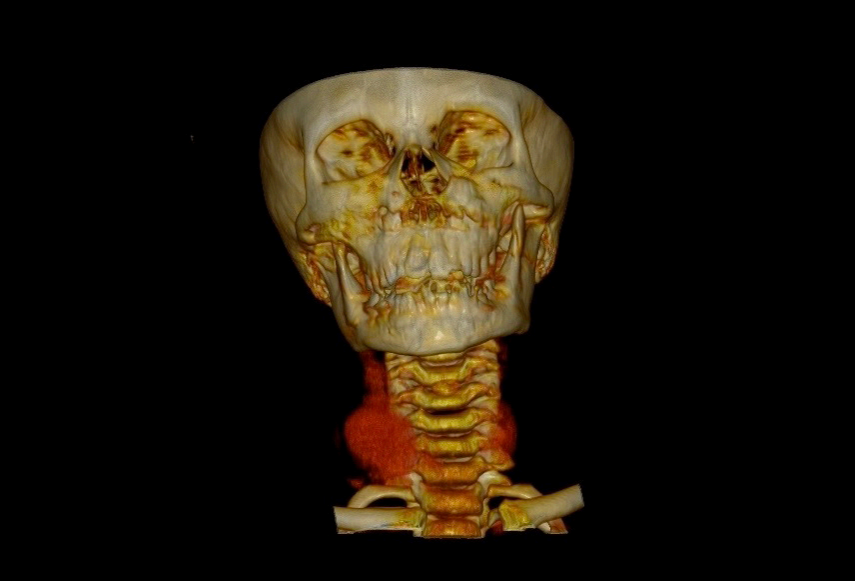
Cervical CT in coronal plane with 3D vascular reconstruction.

3D reconstruction of the CT images was ordered to improve visualization of the dilatations and their relationships to the adjacent structures (Figure 3). The conservative management option was chosen and the patient was discharged from hospital with instructions to return if clinical signs emerged or, if there were no symptoms, to return in up to 1 year for outpatient follow-up with the vascular surgery team.

This is a descriptive study with a case report design. Data were collected from articles published from 1928 to 2023 from the LILACS and MEDLINE databases in order to compare the present case with the literature thus found.

## DISCUSSION

A literature review was conducted to contextualize the findings of the case report described here. Internal jugular phlebectasia can be congenital, idiopathic, or caused by trauma, prior surgery, or tumors. The condition predominantly affects the right internal jugular vein, as described by Bansal, who suggested that intrathoracic pressure is transmitted more to the right jugular vein than to the left. Cases with left unilateral involvement or bilateral involvement are less frequent.^14^

The present case report describes a 6-year-old male patient, which is in line with the literature, which reports greatest prevalence among male pediatric patients. Studies such as those revised by Bansal highlight this predominance.^14^ In the case described, bilateral phlebectasia was observed with greater dilatation on the right side. While the literature describes the most common form as unilateral presentation with a predilection for the right side, bilateral presentations, while rare, have been documented and suggest the possibility of congenital etiology. This hypothesis is supported by the enlargement observed in both jugular veins, which makes an exclusively traumatic origin less likely. However, it was not possible to determine whether the changes were already present before the trauma.^15^

The diagnosis was confirmed by ultrasonography with Doppler and computed tomography (CT), as described in the literature. Doppler ultrasonography is effective for visualization of venous flow and the relationship between the mass and adjacent structures, while CT helps to supplement the diagnosis, yielding a detailed three-dimensional view.^16^ Although internal jugular phlebectasia is considered a benign condition, the literature reports serious complications, such as spontaneous rupture, thrombophlebitis, and pulmonary thromboembolism.^11,16^ Differential diagnoses that should be ruled out in internal jugular phlebectasia cases include branchial cyst, cavernous hemangioma, superior mediastinal cyst, cystic hygroma, and laryngocele.^12^

In the case of venous aneurysms, management can be conservative, with follow-up to monitor progression of the aneurysm, or surgical management can be chosen.^17^ In the present case, the therapeutic approach chosen was conservative, which is in line with what guidelines recommend for asymptomatic cases.^18^ For symptomatic patients, the literature suggests surgical interventions, such as resection of the dilated segment or use of polytetrafluoroethylene (PTFE) grafts.^19^ The risks associated with surgery, such as venous congestion, nerve damage, and thrombosis, highlight the importance of choosing conservative management when there are no complications, although monitoring is still recommended in this situation.^20^ In the case described, the choice to monitor the patient without surgical intervention was appropriate, considering the absence of signs of thrombosis, severe symptoms or imminent risk of rupture. Questions remain with regard to factors such as expansion and dilatation.

The rarity of this condition could be associated with a lack of knowledge among physicians and the tendency to only report surgical cases.^13^ The literature review identified around 100 cases reported globally, with few bilateral cases described in Brazil. In this scenario, the present report contributes to the knowledge base on internal jugular phlebectasia, highlighting its clinical characteristics, diagnostic challenges, and treatment approach in a rare scenario with bilateral involvement.

## CONCLUSIONS

Internal jugular phlebectasia is a benign clinical condition, with a non-tortuous aneurysmal character, involving the internal jugular vein. In our case report, phlebectasia was present bilaterally, but anteroposterior and lateral-lateral diameters were larger on the right side. As described in the literature, conservative management was chosen in the absence of symptoms or complications such as compression of adjacent structures or thrombotic events. Notwithstanding, the dearth of specific studies on the condition highlights the need for additional investigations to improve understanding of its possible complications and to refine diagnosis and definition of therapeutic management.

## Data Availability

Dados não informados ou não utilizados: “Compartilhamento de dados não se aplica a este artigo, pois nenhum dado foi gerado ou analisado”.
