# Supplementary material for: Internal jugular phlebectasia: case report and literature review
Source: J Vasc Bras. 2025 Oct 20;24:e20240095. doi: 10.1590/1677-5449.202400952 (PMC12642931; doi:10.1590/1677-5449.202400952)
Supplement: Translation

# Translation: Flebectasia jugular interna: relato de caso e revisão bibliográfica

**DOI:** 10.1590/1677-5449.202400951

**Authors:** Letícia Stefani Pacheco, Marcos Antonio Bonacorso Manhanelli, José Augusto Costa

**Affiliations:** 1 Pontifícia Universidade Católica de São Paulo – PUC-SP, Faculdade de Ciência Médicas e da Saúde, Sorocaba, SP, Brasil.; 2 Conjunto Hospitalar de Sorocaba – CHS, Sorocaba, SP, Brasil.

**Keywords:** veias jugulares, dilatação, ectasia vascular

## Abstract

A flebectasia jugular interna é a dilatação anormal não tortuosa da veia jugular interna que forma uma massa cervical de caráter aneurismático sacular ou fusiforme, mais incidente em pacientes pediátricos do sexo masculino. As etiologias englobam trauma cervical, neoplasias ou cirurgias prévias. O quadro clínico geralmente é assintomático. Nos casos sintomáticos, a queixa mais frequente é dor na área cervical afetada. O diagnóstico é dado pela visualização da dilatação desencadeada pelo aumento da pressão intratorácica em ações como tossir ou chorar, sendo complementado por exames de imagem como a ultrassonografia com Doppler e a tomografia computadorizada. A conduta terapêutica é conservadora, sendo cirúrgica apenas na presença de queixas ou complicações.

## INTRODUÇÃO

A flebectasia jugular interna pode ser caracterizada como uma condição vascular rara de dilatação não tortuosa anômala da veia jugular interna na área supraclavicular, ao longo da borda anterior do músculo esternocleidomastoideo^1^.

A flebectasia do sistema venoso jugular e cervical é uma condição descrita esporadicamente na literatura, com poucas descrições até a década de 1970. Com o avanço das grandes cirurgias laríngeas e cervicais e com o desenvolvimento de métodos diagnósticos não invasivos, a condição está sendo cada vez mais sendo reconhecida. Mais de 100 casos de flebectasia envolvendo as veias do pescoço, incluindo as veias jugulares anterior e externa, foram relatados na literatura mundial até o momento. O aneurisma venoso foi documentado pela primeira vez em 1928 por Harris, e, em 1952, Gerwig utilizou o termo “flebectasia” para caracterizar essa condição^2-6^. No Brasil, foram relatados casos no intervalo de 1999 a 2021^7-9^.

A maioria dos pacientes são pediátricos do sexo masculino. Clinicamente, a flebectasia jugular interna é assintomática, podendo ser percebida como uma massa de consistência flácida e indolor na lateral do pescoço, comumente de modo unilateral, formando um abaulamento na presença de esforços que aumentem a pressão intratorácica^10,11^.

Pode haver dor cervical associada, além de outras manifestações como alteração na voz, paralisia das cordas vocais e disfagia. Isso ocorre devido à proximidade da veia ao nervo vago e a outros nervos cranianos que podem ser comprimidos pela flebectasia, acarretando na sensação de aperto, sufocamento e vertigem, dor lingual, afonia ao falar alto e desconforto ao praticar atividade física, deglutir ou tossir^12,13^.

## PARTE I - SITUAÇÃO CLÍNICA

Este relato de caso foi aprovado pelo Comitê de Ética em Pesquisa (CEP) sob o CAAE 75131623.5.0000.5373 e o Parecer 6.592.364.

Um paciente de seis anos de idade do sexo masculino foi conduzido ao pronto-socorro após envolvimento em colisão automobilística de média velocidade, com impacto na região lateral direita do pescoço e da cabeça. No pós-trauma imediato, apresentava apenas dor, controlada por analgesia. No exame físico inicial, notou-se abaulamento cervical direito durante o choro, simulando a manobra de Valsalva. O abaulamento era indolor, transitório, extenso, volumoso e de consistência amolecida. A responsável legal pela criança relatou não haver naquele local qualquer massa ou abaulamento antes do trauma.

## PARTE II – CONDUTA

Diante da entrada do paciente na sala de emergência após trauma por acidente automobilístico, solicitou-se, como primeira escolha, uma tomografia computadorizada (TC) de pescoço com contraste (Figuras 1 e 2). O exame confirmou a presença de flebectasia da veia jugular interna, bilateral e mais acentuada à direita. Porém, artefatos de movimento prejudicaram a análise dos cortes.

**Figura 1:** TC com contraste endovenoso em plano axial mostrando veia jugular interna direita.

**Figura 2:** TC com contraste endovenoso em plano axial mostrando veia jugular interna esquerda.

Após a internação, foi realizada a ultrassonografia com Doppler, que demonstrou uma veia jugular interna direita compressível, pérvia, com fluxo fásico à respiração, sem conteúdo ecogênico ou sinais de refluxo patológico. Na TC foram identificadas uma dilatação na veia jugular interna direita (Figura 1) com 2,42 cm no diâmetro ântero-posterior por 3,54 cm no diâmetro látero-lateral, bem como outra dilatação na veia jugular interna esquerda (Figura 2) com 1,60 cm no diâmetro ântero-posterior por 1,32 cm no diâmetro látero-lateral.

Também foi realizada a reconstrução da TC em 3D para melhor visualização da dilatação e de sua relação com as estruturas adjacentes (Figura 3). Optou-se pela conduta conservadora, e o paciente teve alta hospitalar. O paciente foi orientado a retornar caso apresentasse sinais clínicos justificáveis ou, na ausência destes, em até um ano para seguimento ambulatorial com a equipe de cirurgia vascular.

**Figura 3:** TC cervical em plano coronal com reconstrução vascular em 3D.

Este é um estudo descritivo do tipo relato de caso. Foram coletados dados de artigos publicados entre 1928 e 2023 nos bancos de dados LILACS e MEDLINE a fim de comparar o caso descrito com a literatura encontrada.

## DISCUSSÃO

A partir do relato de caso descrito, conduziu-se uma revisão de literatura para contextualizar os achados. A flebectasia jugular interna pode ser congênita, idiopática ou causada por trauma, cirurgias prévias ou tumores. A condição apresenta predominância na veia jugular interna direita, conforme descrito por Bansal, que sugeriu que a pressão intratorácica é mais transmitida à veia jugular direita em comparação à esquerda. Casos de acometimento unilateral esquerdo e de bilateralidade são menos frequentes^14^.

O caso relatado descreve um paciente masculino de seis anos de idade, o que está em conformidade com a literatura, que identifica maior prevalência em pacientes pediátricos do sexo masculino. Estudos como os revisados por Bansal reforçam essa predominância^14^. No caso descrito, foi observada flebectasia bilateral com maior acentuação no lado direito. Embora a literatura descreva a apresentação unilateral, preferencialmente do lado direito, como a forma mais comum, a bilateralidade, apesar de rara, é documentada e sugere uma possível etiologia congênita. Essa hipótese é fortalecida pelo aumento observado em ambas as veias jugulares, o que torna menos provável uma origem exclusivamente traumática. Contudo, não foi possível determinar se essa alteração já existia antes do trauma^15^.

O diagnóstico foi confirmado por ultrassonografia com Doppler e tomografia computadorizada (TC), conforme descrito na literatura. A ultrassonografia com Doppler é eficaz para avaliar o fluxo venoso e a relação da massa com estruturas adjacentes, enquanto a TC auxilia na complementação diagnóstica, permitindo uma visão tridimensional detalhada^16^. Apesar de a flebectasia jugular interna ser considerada uma condição benigna, a literatura aponta complicações graves, como ruptura espontânea, tromboflebite e tromboembolismo pulmonar^11,16^. Alguns diagnósticos diferenciais para a flebectasia jugular interna incluem o cisto branquial, hemangioma cavernoso, cisto mediastinal superior, higroma cístico e a laringocele^12^.

No caso de aneurismas venosos, o manejo pode ser realizado de modo conservador, com o acompanhamento da evolução do aneurisma, ou abordado cirurgicamente^17^. A conduta terapêutica foi conservadora, o que segue as diretrizes recomendadas para casos assintomáticos^18^. Para pacientes sintomáticos, a literatura indica intervenções cirúrgicas, como ressecção do segmento dilatado ou uso de enxertos de politetrafluoretileno (PTFE)^19^. Os riscos associados à cirurgia, como congestão venosa, lesão nervosa e trombose, reforçam a importância de optar pelo manejo conservador na ausência de complicações, situação em que se recomenda manter a monitorização^20^. No caso descrito, a escolha por acompanhamento sem intervenção cirúrgica foi adequada, considerando a ausência de sinais de trombose, sintomas graves ou risco iminente de ruptura. O questionamento permanece frente a fatores como a expansão da dilatação.

A raridade dessa enfermidade pode estar associada à falta de conhecimento entre médicos e à tendência de relatar apenas casos cirúrgicos^13^. A revisão identificou cerca de 100 casos relatados globalmente, sendo poucos os casos bilaterais descritos no Brasil. Nesse contexto, o presente relato contribui para a ampliação da base de conhecimento sobre a flebectasia jugular interna, destacando suas características clínicas, desafios diagnósticos e abordagem terapêutica em um cenário raro de bilateralidade.

## CONCLUSÃO

A flebectasia jugular interna é uma condição clínica benigna, de caráter aneurismático, não tortuoso, da veia jugular interna. No nosso relato de caso, a flebectasia encontrava-se presente bilateralmente, mas com diâmetro ântero-posterior e látero-lateral maior do lado direito. Conforme descrito na literatura, optou-se por conduta conservadora diante da ausência de sintomas ou complicações como compressão de estruturas adjacentes ou eventos trombóticos. Contudo, a escassez de estudos específicos sobre essa condição ressalta a necessidade de mais investigações para melhor compreensão de suas possíveis complicações e para o aprimoramento do diagnóstico e da definição da conduta terapêutica.

## Data Availability

Data not reported or used: “Data sharing does not apply to this article, as no data were generated or analyzed”.
